# Late-Life Presentation of Unsuspected G6PD Deficiency

**DOI:** 10.1155/2018/8198565

**Published:** 2018-09-25

**Authors:** Marcos Benchimol, Laura Bernardo Madeira, Ricardo de Oliveira-Souza

**Affiliations:** ^1^Hospital Universitário Clementino Fraga Filho (UFRJ), Rio de Janeiro, Brazil; ^2^The D'Or Institute for Research & Education (IDOR), Rio de Janeiro, Brazil; ^3^The Federal University of the State of Rio de Janeiro (UNI-RIO), Rio de Janeiro, Brazil

## Abstract

Deficiency of glucose-6-phosphate dehydrogenase (G6PD) is the commonest enzyme deficiency in humans with a wide range of possible clinical manifestations depending on the specific genetic variant in each case. Here we present the case of an 86-year-old male of African descent who acutely developed symptoms of G6PD deficiency immediately after he received methylene blue for treating methemoglobinemia. The contrast between a low SO_2_ on pulse oximetry and a normal arterial gas sampling raised the possibility of methemoglobinemia. The patient was treated with packed red blood cells and folic acid, and a rapid clinical improvement followed by normalization of the red blood cell count ensued. In view of the patient's advanced age, the lack of a history of similar episodes in the past, and the normal laboratory results during the hemolytic crisis, this case remained a diagnostic challenge for over three months, when a follow-up measure of G6DP activity eventually confirmed the diagnosis. A latent deficiency of G6PD may become clinically manifest under the appropriate triggering conditions even in elderly patients and in the absence of past or current clinical and laboratory evidence of G6PD deficiency.

## 1. Introduction

Glucose-6-phosphate dehydrogenase (G6PD) deficiency is the commonest enzyme deficiency in humans, especially in those of African descent [1], with a prevalence of 400 million affected people worldwide [2]. G6PD is polymorphic with more than 300 variants. Beutler reviewed the history, clinical manifestations, genetics, and molecular biology of this disorder [3]. The clinical phenotype is largely dictated by the severity of the enzyme deficiency which, in turn, is determined by the specific genetic variant of G6PD in each case. Accordingly, the clinical manifestations of G6PD may range from none to (i) erythroblastosis fetalis, chronic hemolysis, and (iii) acute hemolytic crisis [4]. Acute hemolytic crises, in particular, are usually caused by exposure to certain medications, systemic infections, and the consumption of fava beans [5, 6]. Here we report on the case of a patient in whom the first manifestations of G6PD deficiency occurred at age 86. Written informed consent was obtained from the patient for publication of the present communication. Data used to support the findings of this presentation have been included in the body of the article.

## 2. Case Presentation

An 86-year-old retired male of African-Brazilian descent was admitted to the Clementino Fraga University Hospital for surgical correction of lumbar stenosis. He had a history of chronic arterial hypertension, stage 3 chronic kidney disease, benign prostatic hyperplasia, peripheral arterial disease, and arthrosis of the knees. He had long been treated with enalapril, hydrochlorothiazide, nifedipine, aspirin, simvastatin, finasteride, cilostazol, and tamsulosin. He had a smoking pack year equal to 30 but had quit smoking several years earlier.

**Figure 1:**
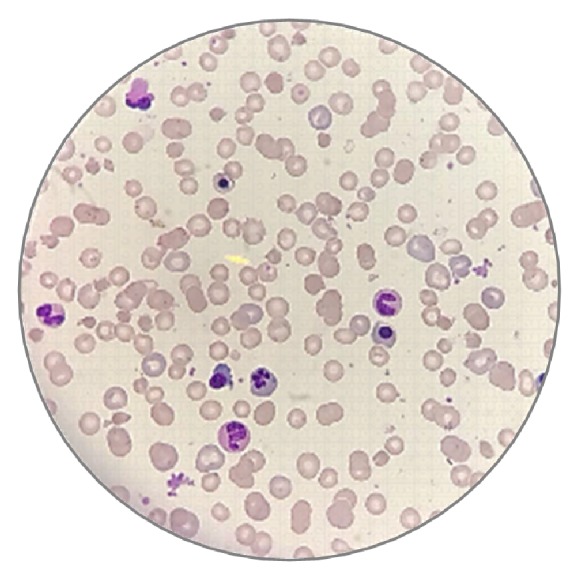
Peripheral blood smear showing polychromatophilia, numerous microspherocytes, and erythroblasts, some of which are dysplastic; schizocytes, Heinz bodies, and “bite cells” were not seen (Giemsa stain).

In the immediate postoperatory period, he developed a hypertensive emergency and was treated with intravenous nitroglycerin. Soon thereafter, he developed cyanosis of the extremities, which was confirmed by pulse oximetry (SO_2_ = 79%), but not by arterial blood gas sampling (SO_2_ = 97%). He was then empirically treated with methylene blue considering the clinical suspicion of methemoglobinemia, but severe dyspnea ensued in close association with the beginning of treatment. His hemoglobin steeply decreased from 11.1 g/dL to 6.1 g/dL, but the physical exam revealed no evidence of bleeding or of liver enlargement. The patient was not aware of previous episodes of anemia. A laboratory work-up revealed an elevated reticulocyte count, macrocytosis, transient leukocytosis (leukocytes count = 14,000/mm^3^ with 60% neutrophils, 30% lymphocytes, 8% monocytes, and 2% eosinophils), normal platelet count (= 250,000/mm^3^), hemoglobinuria, and positive markers for hemolysis [LDH = 4701 U/L (normal < 250 U/L), total bilirubin = 1.6 mg/dL (normal range = 0.3-1.2 mg/dL), unconjugated bilirubin = 0.9 mg/dL (normal < 1 mg/dL), haptoglobin < 6 mg/dL (normal range = 44-215 mg/dL]. A Coombs test was negative. The peripheral blood smear was consistent with a diagnosis of hemolytic anemia (Figure 1). A few days after exposure to methylene blue, the patient's methemoglobin was 4.5% (normal < 2%). Given the possibility that the hemolytic crisis had been triggered by a deficiency of G6PD, a measure of the activity of this enzyme was obtained, resulting in a value of 13.6 U/g Hb (normal > 6.7 U/g Hb). Because enzyme activity was assessed during the hemolytic crisis, a definite diagnosis of G6PD deficiency could not be confidently ruled out at the time.

The patient was treated with packed red blood cells and folic acid. A rapid clinical improvement, which was closely followed by normalization of the red blood cell count, was observed. Three months later, a follow-up measure of G6DP activity (5.5 U/g Hb) revealed a moderate degree of enzyme deficiency (the 10%-60% range), thus confirming the diagnosis of G6DP deficiency.

## 3. Comment

An octogenarian male patient of African descent without current or past evidence of G6PD deficiency developed methemoglobinemia shortly after being treated with venous nitroglycerin. The treatment of methemoglobinemia with methylene blue, in turn, triggered a severe hemolytic crisis and overt symptoms of acute anemia. The contrast between a low SO_2_ on pulse oximetry and a normal arterial gas sampling raised the possibility of methemoglobinemia.

A deficiency of G6PD must be considered in the differential diagnosis of intra- and extravascular hemolysis [7]. An important diagnostic pitfall in like cases is the possibility that the activity of this enzyme may be misleadingly normal because of an elevated reticulocyte count. This may be circumvented by obtaining a new test of enzymatic activity at least three months after the hemolytic crisis has been successfully controlled. A positive genetic test is diagnostic, but its availability and high cost preclude its widespread use.

Saldanha* et al*. [8] found an 8% prevalence of G6DP deficiency in individuals of African descent in São Paulo, Brazil. However, late presentations of G6PD deficiency have seldom been reported. To our knowledge, there is only one case in the literature of a 70-year-old patient who presented with an episode of methemoglobinemia due to type II G6PD deficiency [9]. Several phenotypes of G6PD deficiency have been described and classified according to the magnitude of the enzyme deficiency. Only phenotypes I, II, and III have diagnostic relevance [10]:

- Class I (enzyme activity < 10%): severe G6PD deficiency manifesting clinically as chronic hemolytic anemia
- Class II (enzyme activity < 10%): this phenotype is clinically manifested as intermittent hemolysis, which is usually triggered by exposure to certain drugs or fava beans consumption. Mediterranean G6PD deficiency provides the classical example
- Class III: this phenotype is characterized by a moderate degree (between 10 and 60%) of enzymatic deficiency. Clinically, it gives rise to intermittent hemolytic crises which are usually triggered by oxidative stress. The commonest form of this condition, G6DP-A deficiency, is found in individuals of African descent [11]. The patient presented in this communication fits in this class.

## 4. Conclusion

Caution is recommended in the management of methemoglobinemia triggered by methylene blue treatment. A latent or theretofore asymptomatic deficiency of G6PD may become clinically manifest under the appropriate conditions, especially in patients of African descent, even when evidence of past or current clinical and laboratory evidence of G6PD deficiency is lacking. The contrast between a low SO_2_ pulse oximetry and a normal arterial SO_2_ sampling should raise suspicion on the presence of methemoglobinemia.
